# Decomposing litter and associated microbial activity responses to nitrogen deposition in two subtropical forests containing nitrogen-fixing or non-nitrogen-fixing tree species

**DOI:** 10.1038/s41598-018-30666-5

**Published:** 2018-08-28

**Authors:** Guixiang Zhou, Jiabao Zhang, Xiuwen Qiu, Feng Wei, Xiaofeng Xu

**Affiliations:** 1 Poyang Lake Eco-economy Research Center, Jiujiang University, Jiujiang, 332005 China; 2 State Key Laboratory of Soil and Sustainable Agriculture, Institute of Soil Science, Chinese Academy of Sciences, Nanjing, 210008 China; 3 Jiangxi Agricultural University, Nanchang, 330045 China

## Abstract

Atmospheric nitrogen (N) deposition has caused concern due to its effects on litter decomposition in subtropical regions where N-fixing tree species are widespread. However, the effect of N deposition on litter decomposition in N-fixing plantations remains unclear. We investigated the effects of a 2-year N deposition treatment on litter decomposition, microbial activity, and nutrient release in two subtropical forests containing *Alnus cremastogyne* (*AC*, N-fixing) and *Liquidambar formosana* (*LF*, non-N-fixing). The decomposition rate in *AC* was faster than in *LF* when there was no experimental N deposition. In *AC*, the initial decomposition rate was faster when additional N was applied and was strongly linked to higher cellulose-degrading enzyme activities during the early decomposition stage. However, N deposition reduced litter decomposition and inhibited lignin-degrading enzyme activities during the later decomposition stage. Nitrogen deposition enhanced carbohydrate and alcohol utilization, but suppressed amino acid and carboxylic acid uptake in the *AC* plantation. However, it did not significantly affect litter decomposition and microbial activity in the *LF* plantation. In conclusion, N deposition could inhibit litter decomposition by changing microbial enzyme and metabolic activities during the decomposition process and would increase carbon accumulation and nitrogen retention in subtropical forests with N-fixing tree species.

## Introduction

Litter decomposition is a critically fundamental process that affects nutrient cycles and carbon (C) storage in ecosystems^1^. Plant litter breakdown is affected by the quality of the litter, microbial community composition, soil properties, and the activities and abundance of soil decomposers^2^. The litter nitrogen (N) concentration and soil N availability also have important influences on litter decomposition^2^. Generally, decomposition rates are greater when the initial N concentration is high and the C:N ratio is low^3^.

Fossil fuel combustion, agricultural practices, and the cultivation of N-fixing species have greatly increased the formation and deposition of reactive forms of N^4^. By 2050, global N deposition is expected to have increased to 200 Tg N yr^−1 ^^5,6^. The effects of N addition on litter decomposition have been extensively investigated and the results have shown that there is a high degree of variability in ecosystem responses to N deposition. Several studies have reported that N addition stimulates early stage litter decomposition in N-poor ecosystems by increasing soil N supply or by reducing the litter C:N ratio^7,8^. However, in N-sufficient ecosystems, added N often inhibits litter decomposition. For example, Song *et al*.^9^ found that high-N deposition rates significantly reduced the decomposition of leaf litter in Moso bamboo forest. In N-sufficient ecosystems, supplied N may (1) suppress the synthesis of oxidative enzymes related to lignin degradation^10^; (2) react with polyphenol substrates or compounds produced by microbial decomposition to form more recalcitrant products^11^; and/or (3) change the composition and activity of the microbial communities involved in litter decomposition^12^. In a meta-analysis, Knorr *et al*.^13^ demonstrated that added N had significant positive, neutral, and negative effects on plant litter decomposition. These results indicated that the effect of N deposition on litter decomposition varied considerably because the decomposition of low quality (high lignin or a high C:N ratio) litter types responded negatively to N deposition whereas high quality litters responded positively.

Changes in enzyme and metabolic activities can provide useful insights into the biotic mechanisms underlying microbial sensitivity to N addition. Hydrolytic and oxidative enzymes are two broad groups that respond differently to N deposition. Numerous reports have suggested that the activities of microbial enzymes associated with litter cellulose and lignin degradation are linked to litter decomposition rates in different ecosystems^10,14,15^. Some studies have indicated that the effects of N on decomposition were strongly related to the changes in oxidative enzymes and that these changes correlated with lignin degradation. Carreiro *et al*.^10^ showed that N addition suppressed phenol oxidase activity in high lignin litter, but accelerated or had no significant effect on phenol oxidase in low lignin litter, which was correlated with the effects of N on litter decomposition. In addition, N addition also affects hydrolytic enzymes, such as cellulose-degrading enzymes^10,14,16^. In temperate forests, Sun *et al*.^17^ demonstrated that long-term N addition increased the cellulose-degrading enzyme levels during the early stage of decomposition. The effects of N addition on enzyme activities often varied across sites due to differences in nutrient availability^18^, litter quality^10,19^, and the composition of the microbial community^20^ during the decomposition process.

Nitrogen-fixing tree species are important components of a plantation and are often used as pioneer tree species, especially on degraded and nutrient-poor land^21^. The N-fixing plants can fix the atmospheric N_2_ and improve N availability in soil, resulting in high N concentrations in the plant litters. Previous studies have demonstrated that exogenous N could have inhibitory or neutral effects on plant litter decomposition in N-sufficient forests^11,22^. However, the effect of added N on litter decomposition has not been tested simultaneously in N-fixing plantations and non-N-fixing plantations. This study reports the effects of simulated N deposition on the litter decomposition rate, nutrient release by the litter, and the enzyme and metabolic activities of decomposers in two subtropical plantations containing *Alnus cremastogyne* (*AC*, N-fixing tree species) or *Liquidambar formosana* (*LF*, non-N-fixing tree species) in China. Our final objective was to link the effect of N deposition on litter decomposition to the effect of N deposition on the microbial activity of decomposers. A 2-year experiment was conducted to test the following hypotheses: (1) N deposition would inhibit the decomposition rate of *AC* litter in the *AC* plantation and increase the decomposition rate of *LF* litter in the *LF* plantation; and (2) N addition would suppress the cellulose-degrading and oxidative enzymes in the *AC* plantation and enhance these enzymes in the *LF* plantation.

## Results

### Soil properties and initial litter chemistry

There were significant differences between the *AC* and *LF* plantations for all soil characteristics (*P* < 0.05). Soil organic matter and total N in *AC* were higher than in *LF*, whereas the total P and pH value were lower in *AC* than in *LF* without experimental N addition (Table 1). Nitrogen deposition had no significant effect on soil organic matter and the pH value in *AC* and *LF* (*P* > 0.05, Table 1). Soil total N concentrations increased at the high N addition rate (N2) in both *AC* and *LF* (*P* < 0.05), whereas low N addition (N1) had no significant effect on soil total N in *LF* (*P* > 0.05, Table 1). Nitrogen addition significantly increased the soil total P concentration in *AC*. However, high N deposition (N2) decreased the soil total P concentration in the *LF* plantation (Table 1).

**Table 1:** Soil properties (0–10 cm depth) in the *Alnus cremastogyne* (*AC*) and *Liquidambar formosana* (*LF*) plantations.

Plantation	Treatment	Organic matter (%)	TN (g kg^−1^)	TP (mg kg^−1^)	pH
*AC*	N0	11.43 ± 0.21a	0.57 ± 0.06c	1.17 ± 0.06c	6.17 ± 0.06a
	N1	10.57 ± 0.31a	1.67 ± 0.32b	1.53 ± 0.06b	6.00 ± 0.10a
	N2	10.97 ± 0.55a	2.57 ± 0.06a	2.13 ± 0.15a	6.10 ± 0.00a
*LF*	N0	9.23 ± 0.55ab	0.43 ± 0.06b	1.37 ± 0.06b	6.67 ± 0.15a
	N1	9.77 ± 0.15a	0.67 ± 0.25b	1.83 ± 0.15a	6.63 ± 0.06a
	N2	8.67 ± 0.32b	1.87 ± 0.21a	1.27 ± 0.21b	6.43 ± 0.12a

Soil samples were collected before the beginning of the litter decomposition experiment in August 2015. Organic matter measurements were based on the C concentration and a conversion factor (1.724). *TN*, soil total N; *TP*, soil total P. Values are presented as means ± SE, n = 3. Different lowercase letters indicate significant differences between treatments in each plantation (ANOVA with Tukey’s HSD, *P* < 0.05). N0: without N addition, N1: 30 kg N ha yr.^−1^, N2: 60 kg N ha yr.^−1^ *AC*: *Alnus cremastogyne*, *LF*: *Liquidambar formosana*.

The *AC* litter had significantly lower total K, and C: N and lignin:N ratios than the *LF* litter, but the total C, N, and cellulose contents of the litter were significant higher in *AC* than in *LF* (*P* < 0.01) (Table 2). There were no significant differences between *AC* and *LF* for lignin and total P concentrations (Table 2).

**Table 2:** Initial litter chemistry of the *AC* and *LF* litters.

Litter type	Total C (mg g^−1^)	Total N (mg g^−1^)	Total P (mg g^−1^)	K (mg g^−1^)	Cellulose (mg g^−1^)	Lignin (mg g^−1^)	C:N	Lignin:N
*AC*	456.30 ± 3.56	9.40 ± 0.44	0.29 ± 0.01	1.64 ± 0.05	5.05 ± 0.04	3.82 ± 0.01	48.60 ± 1.92	40.74 ± 2.01
*LF*	418.70 ± 4.51	6.17 ± 0.45	0.32 ± 0.1	2.04 ± 0.07	3.83 ± 0.03	3.71 ± 0.02	71.50 ± 2.97	60.33 ± 4.57
*P*	***	**		**	***		***	***

Values are presented as means ± SE, n = 3. Significance: *P* indicates the differences between the *AC* and *LF* litters, **P < 0.01, ***P < 0.001. *AC*: *Alnus cremastogyne*, *LF*: *Liquidambar formosana*.

### Decomposition rates and patterns

**Figure 1:**
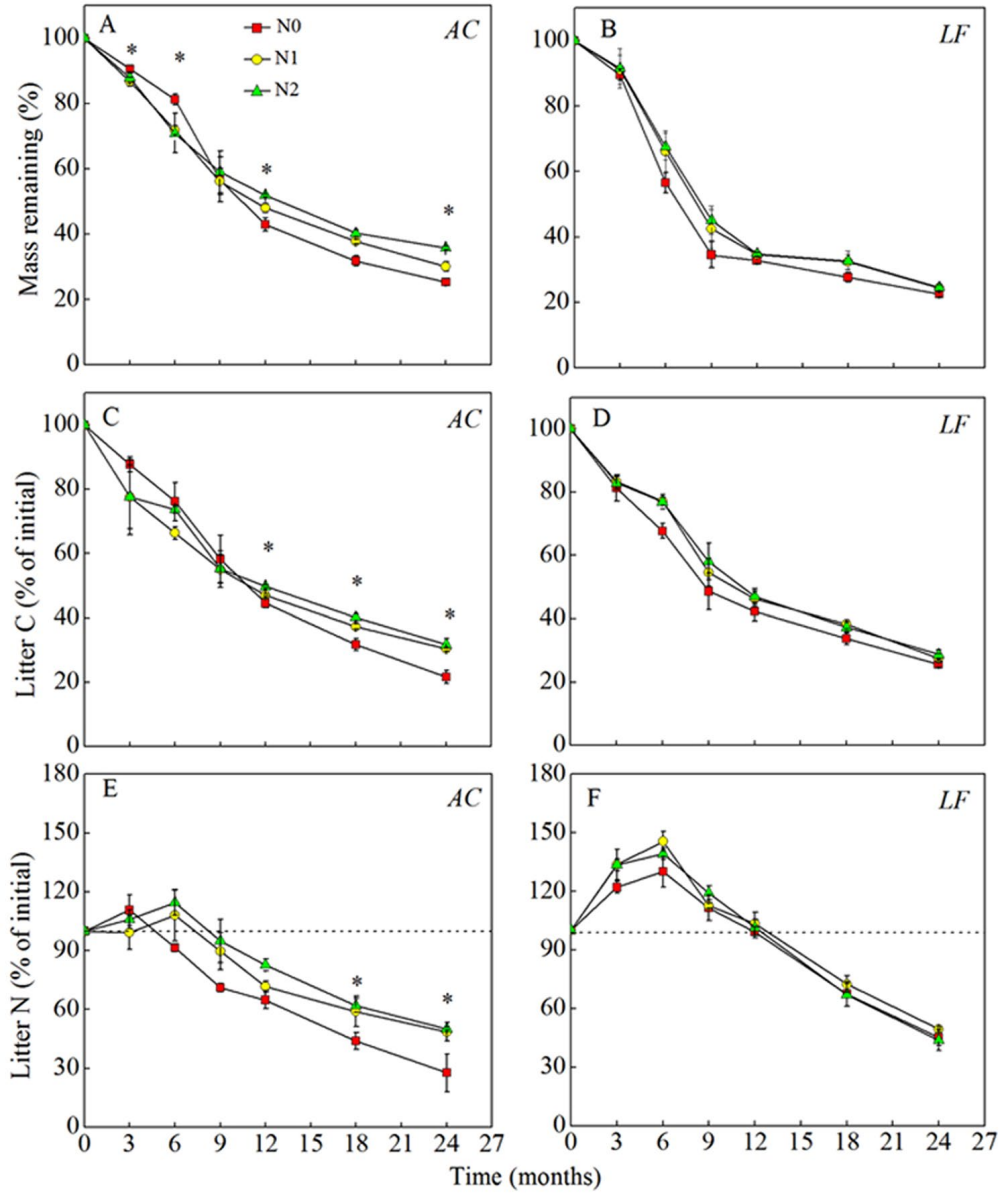
Changes in litter mass loss and nutrient concentrations (% of initial mass) over time for litter decomposition in *AC* (**A**,**C**,**E**) and *LF* (**B**,**D**,**F**) plantations under different N treatments: N0 (control), N1 (30 kg N ha^−1^ yr^−1^), and N2 (60 kg N ha^−1^ yr^−1^). Values are means ± SE. ^*^Indicates significance differences between the control and the N addition treatments at *P* < 0.05. *AC*: *Alnus cremastogyne*, *LF*: *Liquidambar formosana*.

The decay rates (k values) for the litters without N addition (N0) were 0.75 and 0.77 yr^−1^ in *AC* and *LF*, respectively (Fig. S1). Nitrogen deposition significantly decreased the litter decay rates in *AC* (*P* < 0.05) but had no significant effect on the *LF* decay rates (Fig. S1). The single-exponent decomposition model gave good fits for the fraction of the initial mass remaining in the *AC* and *LF* litters (R^2^ values ranged from 0.95 to 0.98 for *AC* (Fig. 1A) and from 0.82 to 0.94 for *LF* (Fig. 1B)).

In the *AC* plantation, litter mass losses were lower in the N addition treatments (N1 and N2) than in the control (N0), especially at 12, 18, and 24 months (Fig. 1A). However, N addition did not significantly affect the *LF* mass loss (Fig. 1B) according to the Tukey’s tests (*P* > 0.05). Similarly, N addition had a negative effect on the proportions of initial litter C and N present during the late stages of decomposition in *AC* (Fig. 1C,E). The ANOVA results showed that N addition had a significant effect on the litter C and N concentrations in the later decomposition stage (*P* < 0.05). However, N addition had no noticeable effect on the proportions of initial C and N remaining in the *LF* plantation (Fig. 1D,F). After 24 months of decomposition, the proportions of the initial mass remaining were 25.2%, 30.1%, and 36.8% for the N0, N1, and N2 treatments, respectively, in *AC*, whereas they were 22.5%, 24.3%, and 24.5%, respectively, in *LF*. The proportions for initial litter C remaining after 24 months were 21.6%, 30.3%, and 31.6%, respectively, in *AC* and 25.7%, 27.5% and 28.7%, respectively, in *LF*.

Both substrates (*AC* and *LF*) showed net immobilization of N in the first 6 months of decomposition. Nitrogen addition promoted N immobilization in the litters compared to the control treatment without N addition (Fig. 1E,F). It also strongly affected the proportional concentration of N in *AC* litter but had no effect on the N concentration in *LF* litter over 24 months (Fig. 1E,F). After 24 months of decomposition, the N concentrations in the *AC* plantation N1 and N2 treatments were 48.7% and 50.2%, respectively, which were much greater than in the N0 treatment (27.7%).

### Enzyme activity

**Figure 2:**
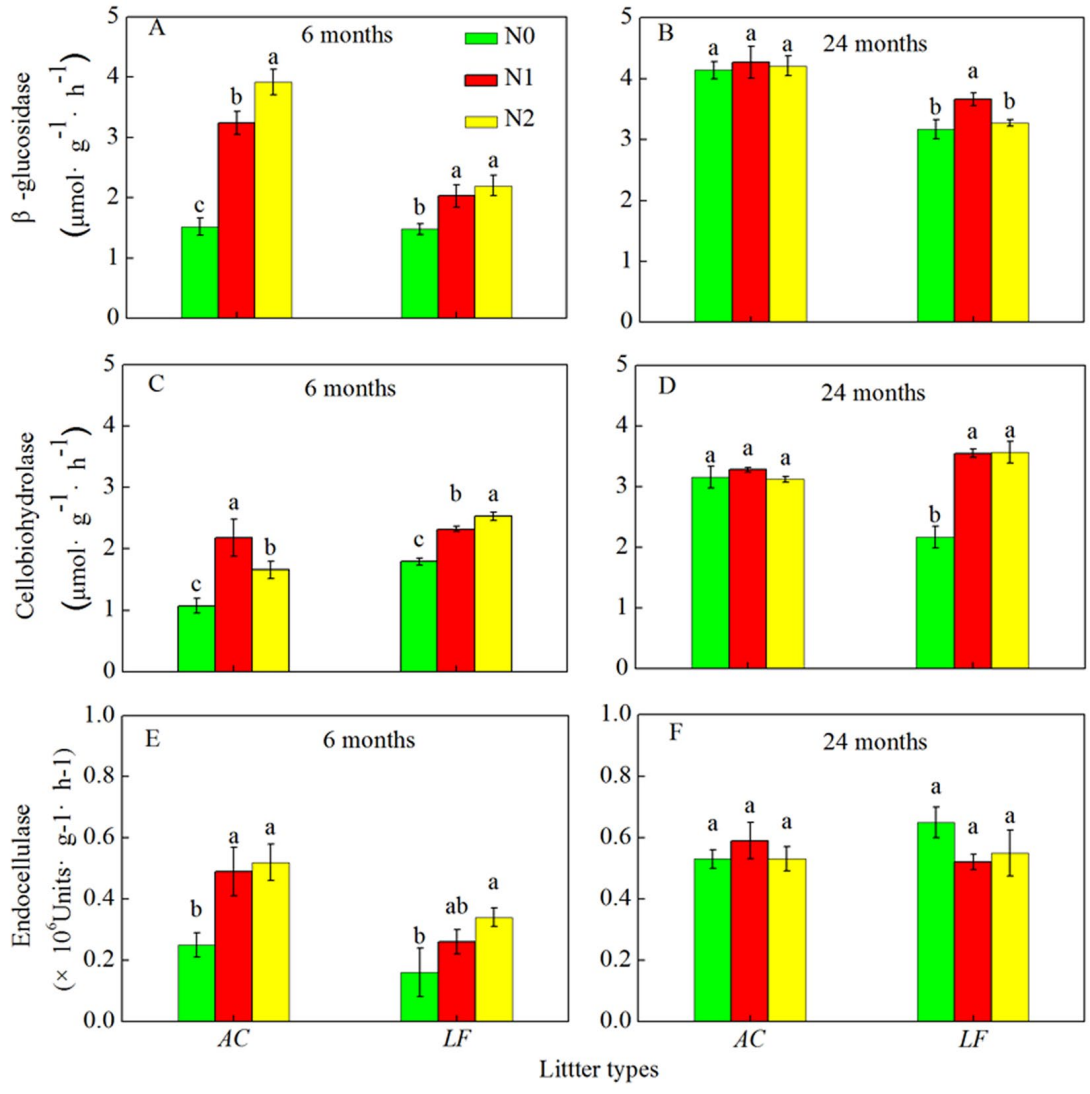
Activities of three cellulose-degrading enzymes (β-glucosidase, cellobiohydrolase, and endocellulase) at different sampling times over a 24 month decomposition period in the N-deposition and control plots. Values are means ± SE. *AC*: *Alnus cremastogyne*, *LF*: *Liquidambar formosana*.

**Figure 3:**
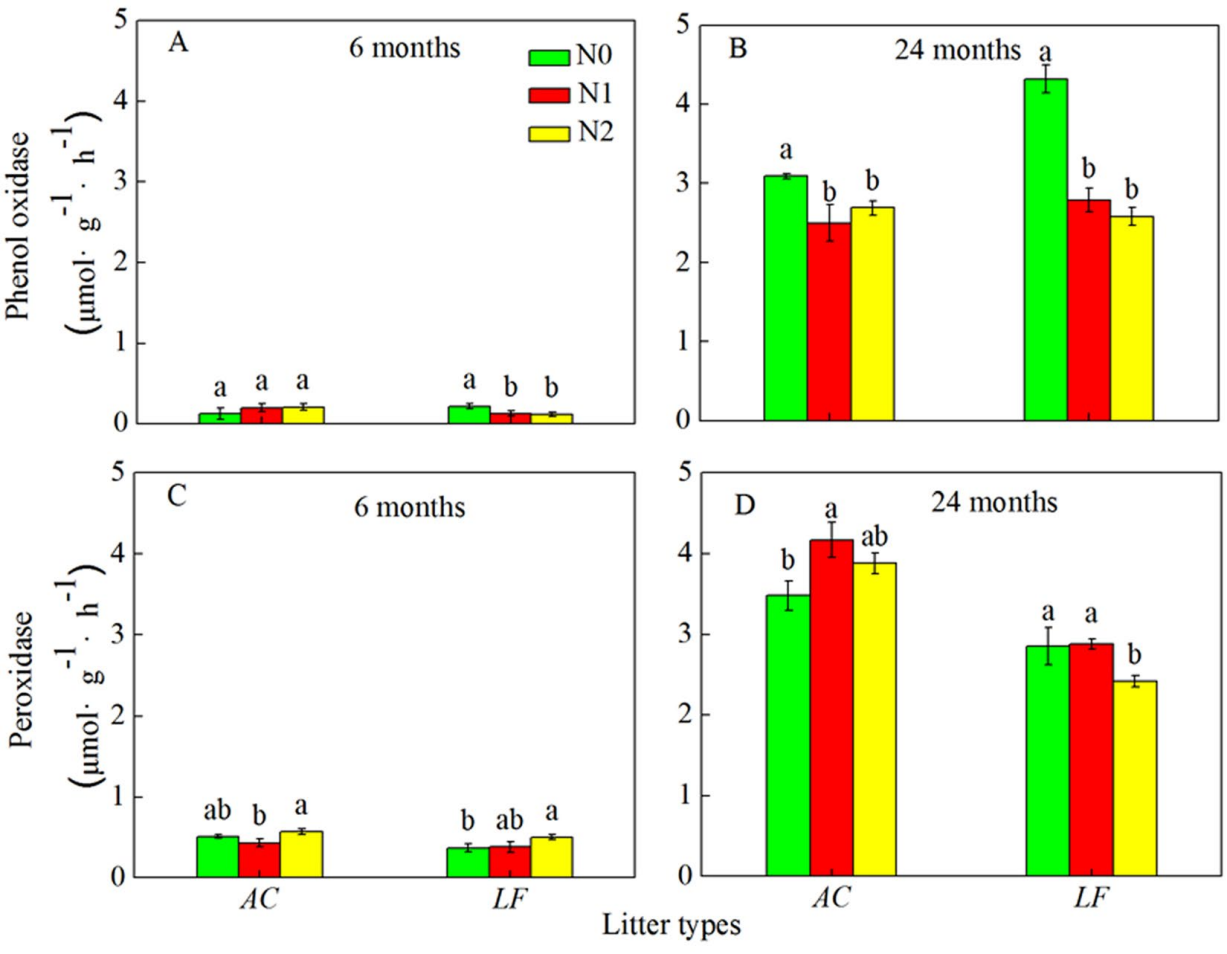
Activities of two lignin-degrading enzymes (phenol oxidase and peroxidase) at different sampling times over a 24 month decomposition period in the N-deposition and control plots. Values are means ± SE. *AC*: *Alnus cremastogyne*, *LF*: *Liquidambar formosana*.

Significant pairwise correlations among the five extracellular enzyme activities were observed because the three cellulose-degrading enzymes correlated with each other (*P* < 0.01 in all cases) across the decomposition period. The two oxidative enzymes also correlated with each other (*P* < 0.01). Across the two litter types, N deposition significantly increased the activities of β-glucosidase, cellobiohydrolase, and endocellulase at the 6-month harvest stage (Fig. 2). After 24 months of decomposition, the effects of N addition on the three cellulose-degrading enzymes were not significant in both the *AC* and *LF* litters, but the cellobiohydrolase activity was significantly increased when N was added to the *LF* litter. The activities of the three cellulose-degrading enzymes increased at 24 months compared to 6 months (Fig. 2). Similarly, the activities of the two oxidative enzymes at 24 months were much greater than they were at 6 months. There was no significant effect of N addition on the activities of the two oxidative enzymes in the *AC* litter at 6 months (Fig. 3). Nitrogen deposition decreased phenol oxidase activity, but increased peroxidase activity in the *LF* litter after 6 months. Furthermore, N addition decreased the activity of phenol oxidase in both the *AC* and *LF* litters after 24 months of litter decomposition. However, added N increased peroxidase activity in the *AC* litter, but decreased its activity in the *LF* litter after 24 months (Fig. 3). Therefore, the effects of N addition on peroxidase activity were related to litter type.

Nitrogen deposition had notable effects on the three cellulose-degrading enzymes and the two oxidative enzymes (Table S1). Enzyme activities also differed among litter types. There were no noticeable differences in cellobiohydrolase activity between the two litter types, whereas there were substantial differences between the litter types for the other enzymes (Table S1). The ANOVA results also showed that the differences in the activities of the enzymes were partly affected by harvest time, and that the activities of the enzymes changed as decomposition progressed (*P* < 0.001, Table S1).

When the enzyme activities in the two litters, the litter C, N, cellulose, and lignin concentrations, and the C:N and lignin:N ratios (Table S2) were analyzed, the results showed that all five enzymes were negatively correlated with the litter C, N, cellulose, and lignin concentrations (*P* < 0.01 in all cases, Table S2). However, there were no relationships between the C:N ratios and the activities of the five enzymes (Table S2). Pairwise correlations among litter characteristics revealed significant correlations between lignin:N and cellobiohydrolase, and phenol oxidase and peroxidase (*P* < 0.05), but there were no correlations between lignin:N and β-glucosidase and endocellulase (*P* > 0.05, Table S2).

### Microbial community metabolic activity

**Figure 4:**
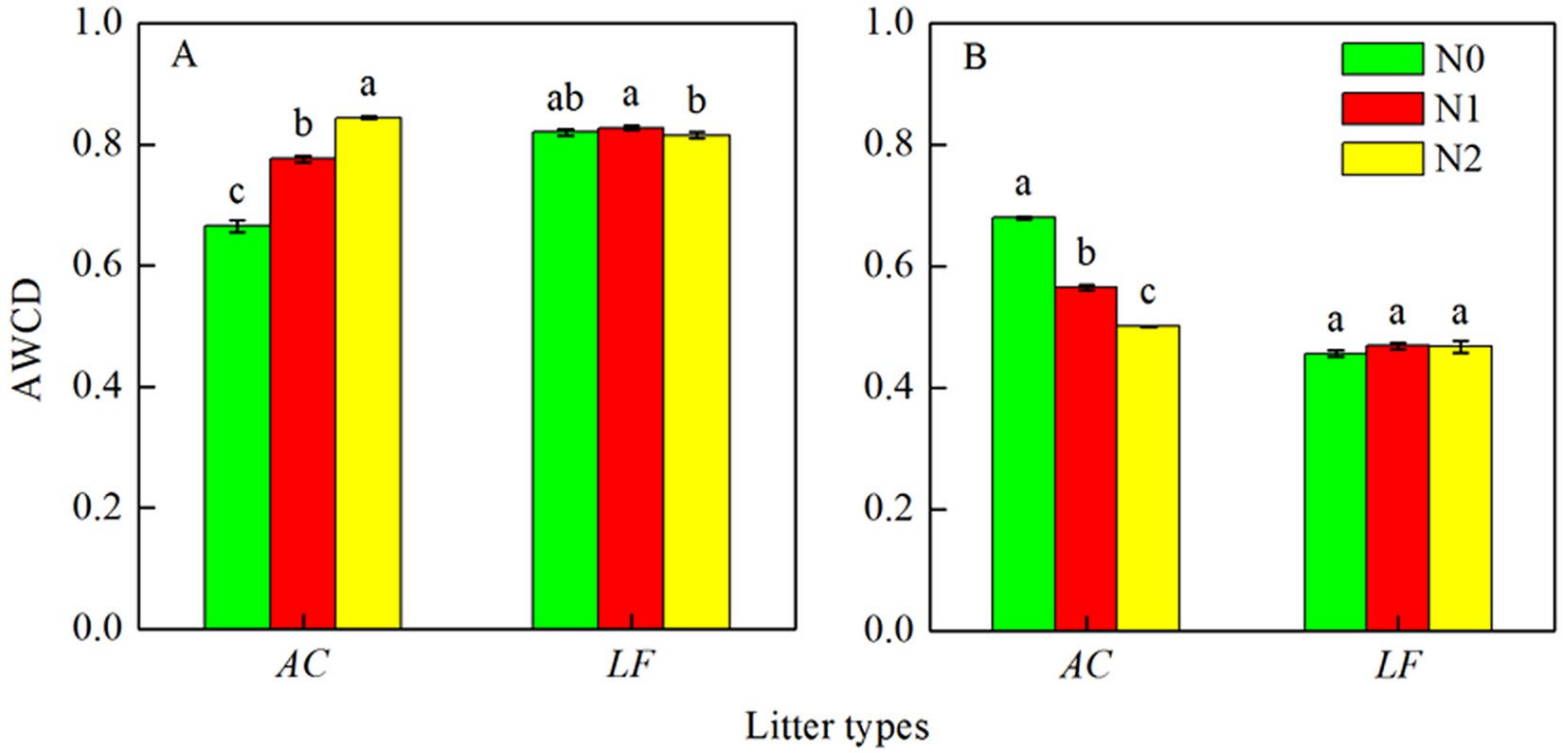
Metabolic activities of the microbial communities and their relationship to litter decomposition when estimated using the Biolog EcoPlates analysis process (Average well color development (AWCD) unit 72 h) at 6 months (**A**) and 24 months (**B**) after N addition. Average well color development data are means ± SE from three replicates. Bars that do not have the same letter (shown above each bar) are significantly different (*P* < 0.05) according to Tukey’s honestly significant difference test. *AC*: *Alnus cremastogyne*, *LF*: *Liquidambar formosana*.

Figure 4 shows the metabolic activities (average well color development, AWCD) of the microbial communities and their relationship with litter decomposition after the Biolog analysis. The results revealed that N addition significantly increased the metabolic activities of the microbial communities in *AC* litter during the early decomposition stage, whereas it did not significantly influence the metabolic activities in *LF* litter. However, added N significantly reduced metabolic activities during the later decomposition stage in *AC* litter. Notably, the metabolic activities of the microbial communities decreased as litter decomposition progressed (Fig. 4).

**Figure 5:**
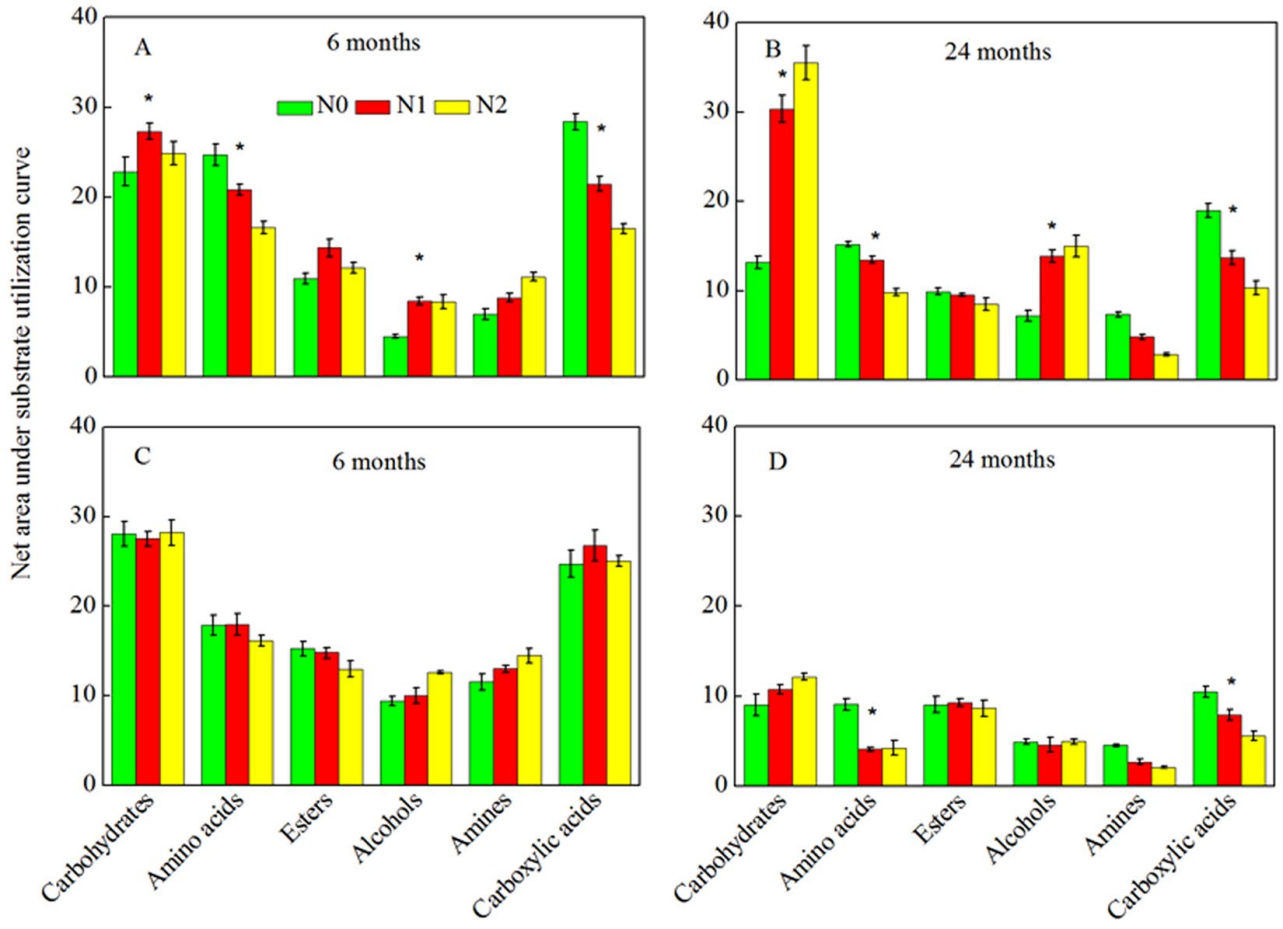
Community-level physiological profiles (CLPP) of *AC* litter (**A**,**B**) and *LF* litter (**C**,**D**) after they had been allowed to decompose for 6 months (**A** and **C**, respectively) and 24 months (**B** and **D**, respectively). *Indicates significance differences between the control and N addition treatments at *P* < 0.05. *AC*: *Alnus cremastogyne*, *LF*: *Liquidambar formosana*.

There were six main categories of substrates. These were classified as carbohydrates, amino acids, esters, alcohols, amines, and carboxylic acids based on their community-level physiological profiles. In the *AC* plantation, the utilization of carbohydrates and alcohols was higher in the N addition treatments than in the control treatment for all decomposition stages, whereas the utilization of amino acids and carboxylic acids was lower in the N addition treatments (Fig. 5). However, utilization of amino acids and carboxylic acids by the microbial communities in the *LF* plantation only declined in the N deposition treatments compared to the control during later decomposition stage. Utilization of the other four categories (carbohydrates, esters, alcohols, and amines) was similar in the different treatments (Fig. 5).

### The effect of litter characteristics and microbial activity on litter decomposition

**Figure 6:**
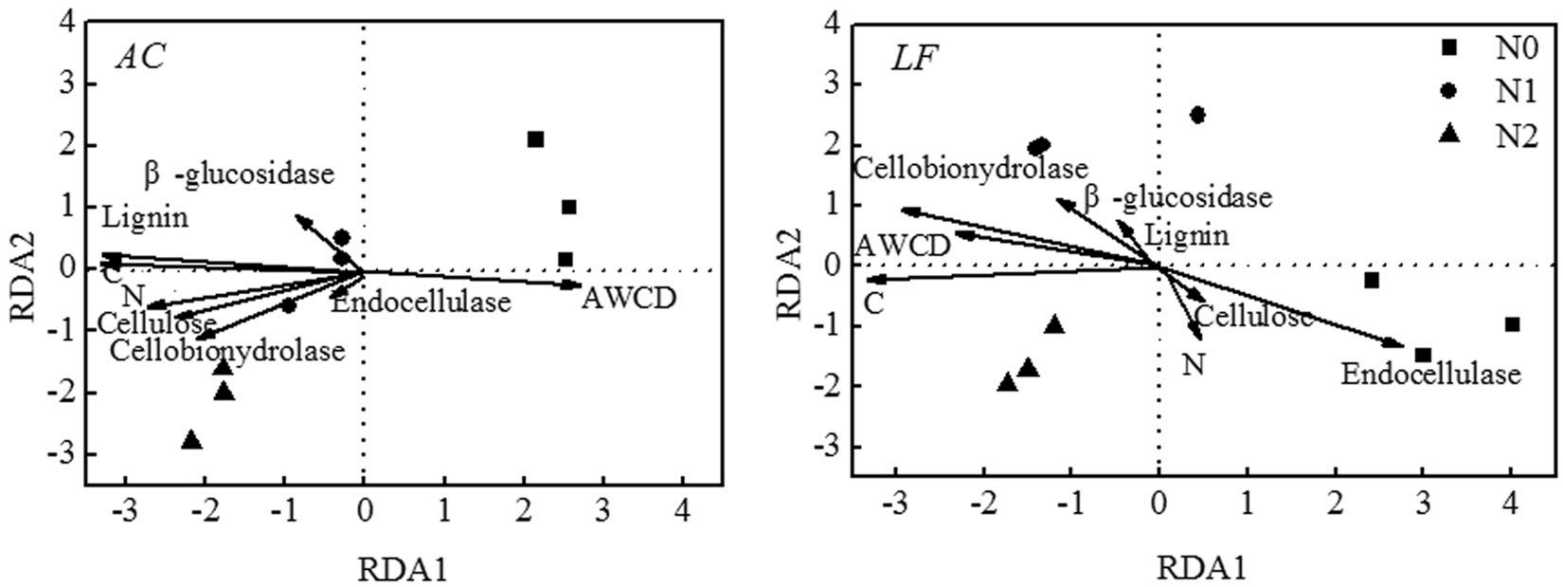
Redundancy analysis (RDA) relating mass remaining to litter quality and microbial activity. *AC*: *Alnus cremastogyne*, *LF*: *Liquidambar formosana*.

A redundancy analyses (RDA) analysis was performed on *AC* and *LF* litters for the whole decomposition period (Fig. 6) in order to separate and evaluate the effects of litter characteristics and microbial community on litter decomposition. The results showed that straw C content was the most important factor affecting litter decomposition in the *AC* and *LF* plantations (Fig. 6). In addition, lignin also had an important effect on litter decomposition in the *AC* plantation (Fig. 6).

### Soil microbial biomass

**Figure 7:**
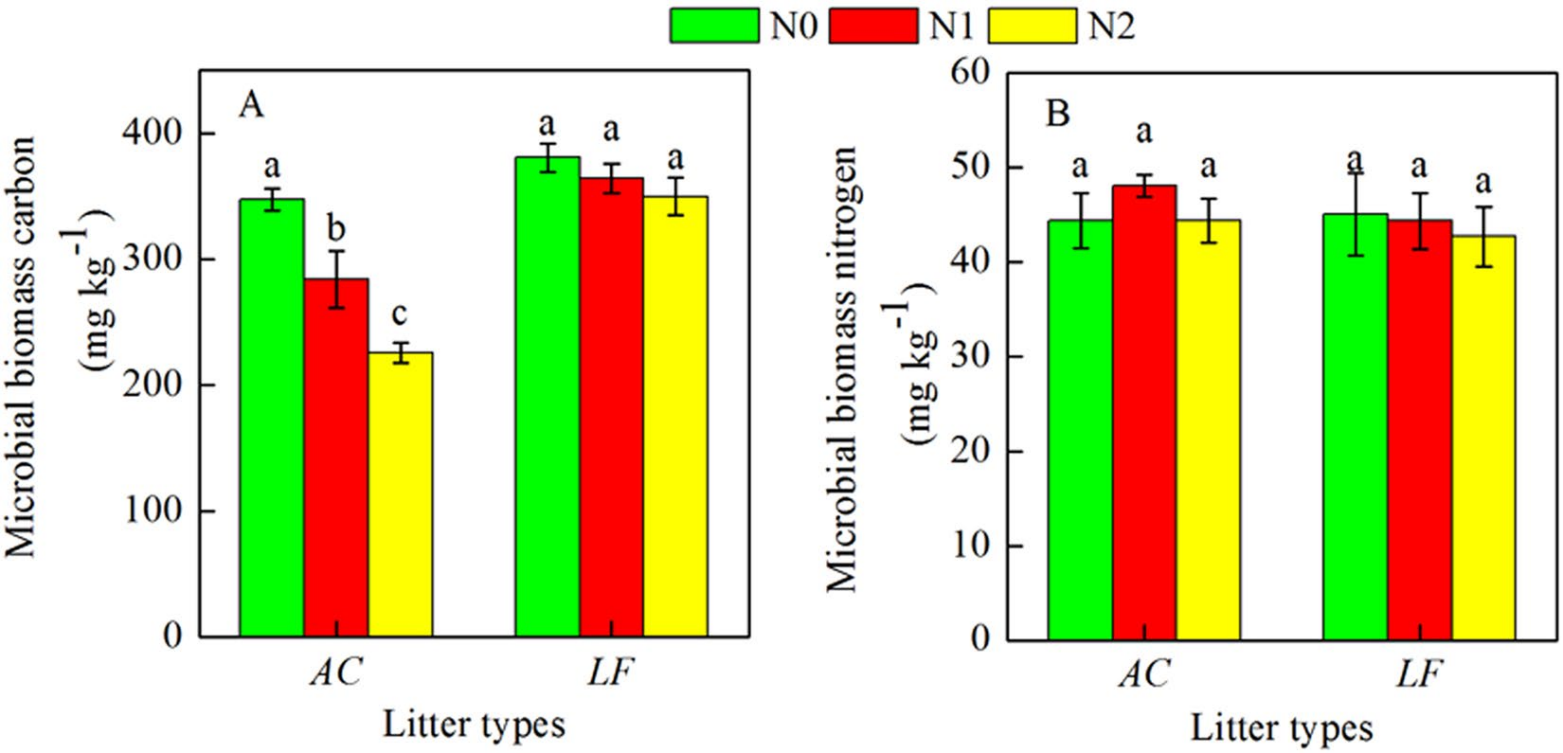
Soil microbial biomass carbon (**A**) and nitrogen (**B**) in the *AC* and *LF* plantations. Soil samples (0–10 cm depth) were collected in August 2017. Different lowercase letters indicate significant differences between treatments in each plantation (ANOVA with Tukey’s HSD, *P* < 0.05). *AC*: *Alnus cremastogyne*, *LF*: *Liquidambar formosana*.

Nitrogen deposition significantly reduced soil microbial biomass C in the *AC* plantation (*P* < 0.001, Fig. 7A). In the *AC* plantation, the mean microbial biomass C concentrations were 347.4 mg C kg^−1^, 283.8 mg C kg^−1^, and 225.4 mg C kg^−1^ in the N0, N1, and N2 plots, respectively. The microbial biomass C in the *LF* plantation also declined after N addition, although the differences were not statistically significant (Fig. 7A). Nitrogen addition did not significantly affect microbial biomass N in either plantation (Fig. 7B).

## Discussion

Over the 24-month decomposition period, N deposition initially speeded up litter decomposition, but the decomposition rate was slower after N addition during the later stage in the *AC* plantation (Fig. 1). A significant inhibitory effect was revealed by the results produced by the single-exponential decay model. Nitrogen addition substantially suppressed the litter decay rate in the *AC* plantation (Fig. S1), which agreed with previous studies^11,17^ and confirmed our first hypothesis. Hobbie *et al*.^1^ also found similar patterns in decomposing *Quercus ellipsoidalis* litter after N addition. However, this effect can vary between different types of litter. In our study, N addition had no significant effect on the mass loss of litter in the *LF* plantation (Fig. 1B), which was inconsistent with our first hypothesis. To our knowledge, few studies have examined the effects of N deposition on litter decomposition in N-fixing and non-N-fixing plantations. The studies that have been undertaken found that the litter decomposition patterns had different responses to N addition and that this was dependent on the species that contributed to the litter^23^. Variable decomposition responses to N deposition by different litter types may improve our understanding of the important role litter quality plays in the decomposition process.

Our results showed that N addition simultaneously increased the activities of cellulose-degrading enzymes, and that N addition accelerated litter decomposition in the *AC* plantation during the early stage. This may be because N addition increased soil N availability to microbes and stimulated the production of enzymes related to cellulose degradation. In addition, an external N supply can adjust the differences in C:N ratios between the microbial community and plant litter, which would change the C use efficiency^17,24^. However, during the later decomposition stage, N deposition generally had no substantial effect on the activities of cellulose-degrading enzymes in both litter types, but there were some exceptions. The lignin-degrading enzyme activity response to N deposition seems to provide some explanations for the inhibitory effects of N addition during the later stages of decomposition in the *AC* plantation. The activity of phenol oxidase, which is produced by white rot fungi and some specialized microbes^25^, was substantially reduced in the N deposition treatments compared to the control in the *AC* plantation during the later decomposition stage. In this study, N addition had a contrasting effect on the *LF* plantation compared to the *AC* plantation. Taken together, these enzyme results were partly consistent with our second hypothesis, and the enzyme activity response to N deposition partially accounts for the effects of N on litter decomposition.

Nitrogen addition may change microbial physiology and community composition, which would ultimately change the litter decomposition rate^18,26^. Our data showed that N addition initially increased the metabolic activities of microbial communities related to litter decomposition, but reduced their metabolic activities during the later decomposition stage in the *AC* plantation. A possible explanation is that N addition increases microbial abundance during the early decomposition stage, but appears to induce the nutritional stress in the later stage. However, N deposition had no significant effect on metabolic activity in the *LF* plantation. These results indicate that the effect of N deposition on the microbial community and its activity partly depends on tree species. Furthermore, it is known that N addition decreases the soil pH, which influences microbial growth and community composition^27,28^. For example, Zhou *et al*.^29^ concluded that soil pH was the dominant factor responsible for the variation in microbial communities across N gradients. Therefore, the variation in metabolic activity may be due to soil pH changes induced by N addition. Nitrogen deposition affects the production of organic N exudates and reduces the litter C:N ratio so that the change in the utilization of amino acids and carboxylic acids fits adaptations made by the microbial community to the increased availability of N^30^. However, metabolic activity did have a significant effect on litter decomposition in the *LF* plantation (Fig. 6). Fisk *et al*.^31^ also found the microbial substrate utilization patterns differed in shrub (dominated by *Chamaedaphne calyculata*) sites and forest (dominated by *(Acer rubrum*, *Pinus strobus* and *Tsuga canadensis*) sites.

Atmospheric N deposition can also have a direct effect on microbial biomass and activity. Bragazza *et al*.^32^ found that 7 years of N fertilization significantly decreased litter C accumulation in bogs because it altered the bacterial biomass and microbial activities. Our results suggested that N addition significantly reduced soil microbial biomass C (Fig. 7), which could also lead to changes in microbial biomass C:N ratios in the soil. This may imply that a highly efficient, fungus-dominated community with a high C:N ratio could shift to a less efficient, bacteria-dominant community with a low C:N ratio^33^.

The initial litter quality and the stoichiometric requirements of the microbial decomposers are the two main factors regulating the immobilization or mineralization of nutrients from decomposing substrates^34^. Nitrogen addition increased C mineralization during the initial decomposition stage in both *AC* and *LF* litter. During the later stage, high N deposition inhibited the loss of C in *AC*, but did not have any significant effect on C mineralization in *LF* litter (Fig. 2). It has been reported that N release generally occurs when the initial N content ranges from 0.6% to 2.8%^35^ or the C:N ratio is extremely low (less than the critical threshold of 5–15)^24,36^. Microbial decomposers may immobilize N from the environment when decomposing N-poor litter to meet the stoichiometric requirements of the microbes. In this study, N was immobilized in both the *AC* and *LF* litters during the early stage of decomposition, whereas it was released later on in the process. The effects of N addition on the litter nutrient loss varied with decay stage. Nitrogen addition increased the accumulation of N during initial decomposition, but high N deposition inhibited the release of N in *AC* during the later stage. A previous study indicated that extra N inputs also decreased the microbial biomass C:N ratios^23^ leading to a wider gap between the litter C:N ratio and the microbial biomass C:N ratio. Zhu *et al*.^23^ added to growing evidence that the suppressing effect of N deposition on litter N release was greater in N-sufficient litter than N-poor ones because N release was suppressed to a greater degree in *Acacia auriculiformis* (with a high initial N concentration) than in *Eucalyptus urophylla* (with a low initial N concentration) when additional N was supplied, which agreed with our results.

A previous report showed that litter chemistry (such as the C:N and lignin:N ratios) explained the majority of the variation in decomposition rates^37^. This study indicated that the nitrogen effect on high-quality (*AC*, with low C:N and lignin:N ratios) litter decomposition was significantly larger than that on low-quality (*LF*, with high C:N and lignin:N ratios) litter decomposition. A possible reason for this may be that the negative effects of N addition on high-quality litter decomposition is linked to the reaction between inorganic N and lignin, and/or the phenolic compounds, synthesized by decomposers, when they react with inorganic N, which results in the accumulation of more recalcitrant substances^17^. The addition of NH_4_NO_3_ may have accelerated the formation of these recalcitrant compounds, and this led to decreased decomposition in the N deposition treatments compared to the control^38^. In previous studies, N addition reduced the lignin-degrading enzyme activity in the litter^4,17^. Our findings showed that litter C and lignin contents strongly affected litter decomposition in the *AC* plantation (Fig. 6), which partly supports the above statement. Another mechanism for the negative N effect is related to the “nitrogen mining” theory. According to the theory, lignin decomposition is a mechanism that releases N from the cell wall when no nitrogen is added. As a result, microbes need to secret lignin-degrading enzymes to break down lignin and access the protected N. In contrast, N is easily available in mineral form when N is added, which means that microbes reduce their lignolytic enzyme production and have little incentive to decompose lignin under N enrichment conditions^39^. Therefore, high N suppression is expected in high-quality litters. Furthermore, our findings were in line with microbial “N mining” theory.

In conclusion, these results demonstrate that N addition significantly inhibited litter decomposition and suppressed C and N release from litter in the *AC* plantation over a 24-month period. However, N deposition did not significantly affect the litter decomposition process in the *LF* plantation. The *AC* plantation results showed that N addition had inconsistent effects on the activities of different enzymes because it stimulated the activities of cellulose-degrading enzymes and decreased the activities of lignin-degrading enzymes. In addition, N deposition enhanced microbial activity during the early decomposition stage, but reduced microbial activity during the later stage in the *AC* plantation. Nitrogen addition to the *LF* plantation had no significant influence on enzyme activities and microbial metabolic activities during litter decomposition. Results from this study suggest that elevated N deposition may have inconsistent effects on litter decomposition and the dynamics of different soil carbon pools in the *AC* and *LF* plantations. Further research on finer temporal scales is needed to reveal the mechanisms that regulate the long-term effects of N deposition on the decomposition of litter from N-fixing and non-fixing tree species.

## Methods

### Study site characterization

The study was located in Jiujiang City (29°68′N, 115°98′E), Jiangxi Province, China. The mean annual temperature at the experimental site is 17 °C and the mean annual precipitation is 1407 m. The area has a monsoonal subtropical climate with four distinct seasons and an annual average of 240 frost-free days. The local ambient atmospheric N deposition is 30.9 kg N ha^−1^ yr^−1 9^. Two research sites (an N-fixing plantation and a non-N-fixing plantation), which were located 1000 m apart, were established to investigate the effect of N deposition on litter decomposition. The plantations were not subjected to any management practices, such as fertilization and plowing, before this study. A survey conducted in July 2015 (before the first N addition) showed that the dominant tree species in the N-fixing plantation was *A*. *cremastogyne* (*AC*), whereas *L*. *formosana* (*LF*) dominated the non-N-fixing plantation. Other tree species in the two plantations were cut down before the first N addition. The soils in both plantations are classified as Ferrisols derived from granite.

### Experimental treatments

We began the N addition treatments at each site in August 2015. The plots (10 m × 10 m) received one of the following three treatments (three replicate plots per treatment): N0 (control, no nutrient addition), N1 (30 kg N ha^−1^ yr^−1^), or N2 (60 kg N ha^−1^ yr^−1^). A total of 18 plots were established in these two plantations. There were nine plots in the N-fixing plantation and nine in the non-N-fixing plantation. All treatments and plots were randomly established in the two plantations. Each plot was surrounded by a 10 m wide buffer strip. Before the first N addition, one soil sample was collected from each plot, and the three soil samples (one from each replicate) were homogeneously mixed into one sample for each treatment. Three soil samples (0–10 cm depth) were collected from both the *AC* and *LF* plantations in July 2015, and the soil properties are summarized in Table 1. Ammonium nitrate (NH_4_NO_3_) dissolved in 10 L water was sprayed uniformly using a sprayer onto the N1 (71.4 g NH_4_NO_3_) and N2 (142.8 g NH_4_NO_3_) plots at the end of each month. There were 12 equal applications throughout the experimental period. The control plots (N0) received 10 L water without NH_4_NO_3_ at monthly intervals.

### Litter decomposition experiment

Freshly fallen leaf litters from the floor of the *AC* and *LF* control treatment plots were collected in July 2015. The litters were oven-dried at 65 °C to a constant weight in a dryer. The oven-dried litter samples were cut into 1 cm pieces with a chopper. The initial characteristics of the two litter types are summarized in Table 2. Nylon mesh bags (20 cm × 15 cm, mesh size 1 mm) were filled with 15 g of one of the litter types. A total of 162 litterbags containing *AC* leaves were positioned on the surface of the organic horizon in the *AC* plantation, and 162 with *LF* leaves were placed in the *LF* plantation at the beginning of the study in August 2015. Three litterbags were harvested and homogeneously mixed to create one sample per plot at each sample time, and the litterbags were sampled after 3, 6, 9, 12, 18, and 24 months. The litter samples collected at each harvest time were delivered to the laboratory as soon as possible and immediately analyzed for enzyme activity or stored at −20 °C for microbial community analysis. Mineral soils (0–10 cm depth) were collected from all plots in the *AC* and *LF* plantations in August 2017. Three soil samples were chosen randomly and mixed to create one sample per plot. The soil samples were used to determine soil microbial biomass carbon and nitrogen.

### Chemical analyses

The oven-dried litter samples were weighed, ground with a grinder, and sieved. The total C and N concentrations in the litters were determined using a CN analyzer (Elementar, Vario Micro Select, Germany). The P and K concentrations in the litters were determined using a modified Kjeldahl method and a UV spectrophotometer or flamephotometer, respectively^40^. The cellulose and lignin concentrations were determined using the acid-detergent fiber method^9^.

### Enzyme activity

We analyzed the litter samples for the following extracellular enzyme activity based on the methods reported by Saiya-Cork *et al*.^41^ and Sinsabaugh and Sinsabaugh *et al*.^14^: β-1,4-glucosidase, cellobiohydrolase, endocellulase, phenol oxidase, and peroxidase. β-glucosidase, cellobiohydrolase, and endocellulase are involved in cellulose decomposition, and phenol oxidase and peroxidase play roles in lignin decomposition. The substrates used to measure β-glucosidase, cellobiohydrolase, and endocellulase were *p*-nitrophenyl-b-D-glucopyranoside, *p*-nitrophenyl-b-D-cellobioside, and carboxymethylcellulose, respectively. The 0.5 g samples were homogenized in 125 mL of 50 mmol/L acetate buffer (pH = 5.0) in a blender and absorbance was measured at 410 nm using a spectrophotometer. The substrates used to measure phenol oxidase and peroxidase were L-3,4-dihydroxyphenylalanine (L-DOPA), and L-DOPA and hydrogen peroxide, respectively. Phenol oxidase and peroxidase were measured using a microplate spectrophotometer at 460 nm.

### Biolog analysis

The Biolog EcoPlate system (Biolog Inc., Hayward, CA, USA) was used to assess metabolic activity after 6 months and 24 months of N addition. Litter samples (~0.1 g) were resuspended in 49.9 mL sterile physiological saline (0.9% NaCl solution) and homogenized. The solution containing litter and physiological saline was shaken at 150 rpm for 30 min and then the pieces of litter were removed from the solution. A total of 150 μL of the solution was added to each well of the Biolog EcoPlates using a pipette. The plates were incubated in an incubator at 25 °C for 6 days, and absorbance was measured at 590 nm every 24 h. The absorbance data at 72 h showed the largest difference among treatments and therefore was used to calculate the average well color development (AWCD), which represents metabolic activity. The community-level physiological profile (CLPP) was calculated as the net area under the curve according to Olson^42^.

### Data analysis

The litter mass remaining in the bags at the end of the experimental period was expressed as a percentage of the initial straw weight in each litter bag. The single-exponential decay model X_t_/X_0_ = e^−*k*t^ was used to calculate the annual decomposition rate constant *k* (yr^−1^), where X_t_ is the mass remaining at time t, and X_0_ is the initial mass. The nutrient release during litter decomposition was expressed as a proportion of the initial nutrient concentration, which was calculated by determining the nutrient concentration at each harvest time and then dividing it by the initial nutrient content. Nutrient release (%) = 100 × [(M_t_ × C_t_)/(M_0_ × C_0_)], where M_t_ is the mass at time *t*; C_t_ is the nutrient concentration at time *t*; M_0_ is the initial mass; and C_0_ is the initial nutrient concentration.

One-way analysis of variance (ANOVA) and a Tukey’s HSD test were conducted to assess the statistical significance of the differences between the annual decomposition rate, mass remaining, nutrient release, enzyme activity, and AWCD at each sampling time for the litters at the *P* < 0.05 level. A multi-factor ANOVA was conducted to determine the effects of litter type, time, and N addition on enzyme activity. Pearson correlations were used to test the significance of the relationships between enzyme activities and litter characteristics. Redundancy analyses (RDA), which related mass remaining to litter quality, enzyme activity, and metabolic activity, were performed using the Vegan package in R ver. 2.8.1 (R Development Core Team. Vienna, Austria). All data were tested to meet the assumptions of normality and homogeneity of variance. The data was transformed when necessary. The values were expressed as mean ± standard deviation (SE, n = 3). All data were analyzed using SPSS 16.0 (Statistical Package for the Social Sciences) and the figures were drawn by software Origin 8.0.

## Electronic supplementary material

Supplementary information
