# Supplementary material for: Decomposing litter and associated microbial activity responses to nitrogen deposition in two subtropical forests containing nitrogen-fixing or non-nitrogen-fixing tree species
Source: Sci Rep. 2018 Aug 28;8:12934. doi: 10.1038/s41598-018-30666-5 (PMC6113256; doi:10.1038/s41598-018-30666-5)
Supplement: Supplementary file 1 — Supplementary information [file 41598_2018_30666_MOESM1_ESM.pdf]

Supplementary information

Decomposing litter and associated microbial activity responses to nitrogen deposition in two subtropical forests containing nitrogen-fixing or non-nitrogen-fixing tree species

Guixiang Zhou[1,2], Jiabao Zhang[2], Xiuwen Qiu[1*], Feng Wei[3], Xiaofeng Xu[3]

[1] Poyang Lake Eco-economy Research Center, Jiujiang University, Jiujiang 332005, China

[2] State Key Laboratory of Soil and Sustainable Agriculture, Institute of Soil Science, Chinese Academy of Sciences, Nanjing 210008, China

[3] Jiangxi Agricultural University, Nanchang 330045, China

Correspondence and requests for materials should be addressed to X.W.Q. (email: qiuxiuwen5@163.com)

Figure S1. The litter decay rate of *AC* and *LF*. Different lowercase indicated significant difference between treatments at each plantation) (Tukey's HSD, $P < 0.05$).

Table S1. Significances of ANOVA for enzyme activity in two plantations.

Table S2. Significances of relationships between enzymes activities and litter characteristics.

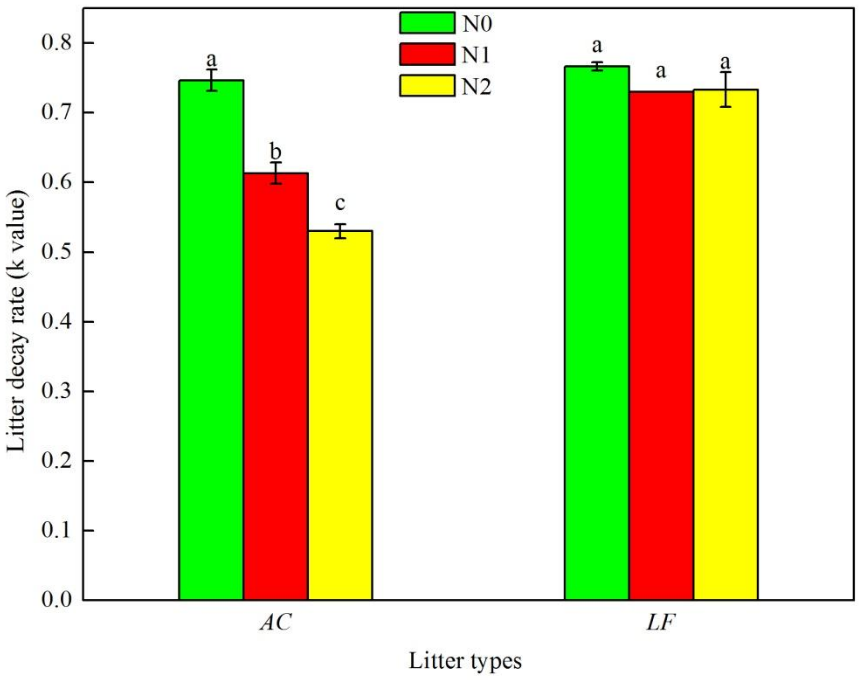

Figure S1. The litter decay rate of *AC* and *LF*. Different lowercase indicated significant difference between treatments at each plantation) (Tukey's HSD, $P < 0.05$).

Table S1. Significances of ANOVA for enzyme activity in two plantations.

Source of variation	β-glucosidase		Cellobiohydrolase		Endocellulase		Phenol oxidase		Peroxidase	
	F	*P*	F	*P*	F	*P*	F	*P*	F	*P*
Type	282.41	0	2.60	0.12	14.12	0.001	37.01	0	234.17	0
Time	642.10	0	874.04	0	130.96	0	6034.18	0	5015.75	0
N	89.72	0	151.00	0	7.23	0.003	96.88	0	6.19	0.007
Type × Time	1.97	0.174	115.02	0	24.53	0	45.21	0	169.39	0
Type × N	19.05	0	5.19	0.013	6.35	0.006	38.90	0	8.20	0.002
Time × N	60.76	0	15.98	0	17.48	0	93.01	0	15.01	0
Type × Time × N	24.59	0	37.25	0	0.15	0.863	21.48	0	12.06	0

Table S2. Significances of relationships between enzymes activities and litter characteristics.

Source of variation	β-glucosidase		Cellobiohydrolase		Endocellulase		Phenol oxidase		Peroxidase	
	F	*P*	F	*P*	F	*P*	F	*P*	F	*P*
Litter C	-0.695	***	-0.734	***	-0.707	***	-0.951	***	-0.924	***
Litter N	-0.676	***	-0.612	***	-0.696	***	-0.886	***	-0.88	***
Cellulose	-0.621	***	-0.709	***	-0.685	***	-0.922	***	-0.83	***
Lignin	-0.445	**0.007	-0.708	***	-0.464	***	-0.892	***	-0.86	***
C:N	0.13	0.451	-0.057	0.74	0.078	0.65	0.085	0.623	0.141	0.412
Lignin:N	0.011	0.951	-0.444	**0.007	-0.052	0.76	-0.466	**0.004	-0.389	*0.019
